# 2-{4-Methyl-*N*-[(2,3,4,9-tetra­hydro-1*H*-carbazol-3-yl)meth­yl]benzene­sulfon­amido}­ethyl 4-methyl­benzene­sulfonate

**DOI:** 10.1107/S1600536813031097

**Published:** 2013-11-20

**Authors:** Mustafa Göçmentürk, Yavuz Ergün, Berline Mougang-Soume, Nagihan Çaylak Delibaş, Tuncer Hökelek

**Affiliations:** a Dokuz Eylül University, Faculty of Sciences, Department of Chemistry, Tınaztepe, 35160 Buca, İzmir, Turkey; b Université de Montréal, Département de Chimie, H3C 3J7, Montréal, Québec, Canada; c Department of Physics, Sakarya University, 54187 Esentepe, Sakarya, Turkey; d Hacettepe University, Department of Physics, 06800 Beytepe, Ankara, Turkey

## Abstract

In the title compound, C_29_H_32_N_2_O_5_S_2_, the indole ring system is nearly planar, with a maximum deviation of 0.013 (2) Å, and the cyclo­hexenone ring has an envelope conformation with the methine C atom as the flap. The two methyl­benzene rings are approximately perpendicular to each other, making a dihedral angle of 89.09 (8)°. In the crystal, N—H⋯O hydrogen bonds link the mol­ecules into a chain running along the *a-*axis direction, and weak C—H⋯O hydrogen bonds and C—H⋯π inter­actions are observed between the chains.

## Related literature

For tetra­hydro­carbazole systems present in the framework of a number of indole-type alkaloids of biological inter­est, see: Saxton (1983 ▶). For related structures, see: Hökelek *et al.* (2009 ▶); Çaylak *et al.* (2007 ▶); Uludağ *et al.* (2009 ▶); Gündoğdu *et al.* (2011 ▶). For the use of tetra­hydro­carbazolone in the synthesis of central-nervous-system-active drugs, see: Romeo *et al.* (2006 ▶). For the syntheses of tetra­hydro­carbazolone-based anti­tumor-active compounds from tetra­hydro­carbazoles, see: Chen *et al.* (2009 ▶). For the syntheses of amino­tetra­hydro­carbazoles as central nervous system agents, see: Mooradian *et al.* (1977 ▶). For bond-length data, see: Allen *et al.* (1987 ▶).
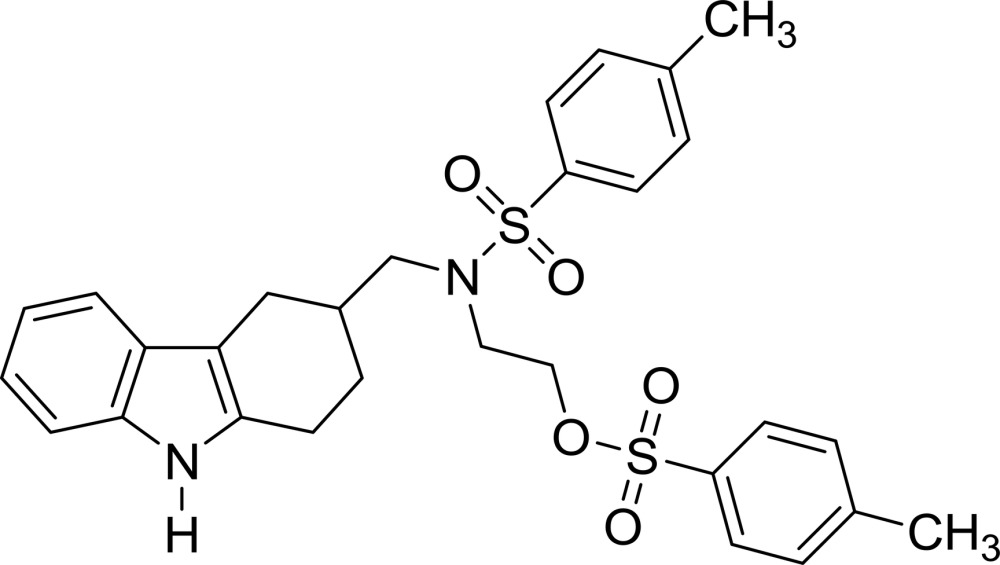


## Experimental

### 

#### Crystal data

C_29_H_32_N_2_O_5_S_2_
*M* *_r_* = 552.69
Monoclinic, 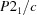

*a* = 10.5719 (5) Å
*b* = 10.8783 (6) Å
*c* = 23.8868 (11) Å
β = 93.507 (2)°
*V* = 2741.9 (2) Å^3^
*Z* = 4
Cu *K*α radiation
μ = 2.11 mm^−1^
*T* = 150 K
0.21 × 0.16 × 0.13 mm

#### Data collection

Bruker Kappa APEXII CCD area-detector diffractometer
Absorption correction: multi-scan (*SADABS*; Bruker, 2005 ▶) *T*_min_ = 0.701, *T*_max_ = 0.761
168526 measured reflections
5101 independent reflections
4955 reflections with *I* > 2σ(*I*)
*R*_int_ = 0.048

#### Refinement

*R*[*F* ^2^ > 2σ(*F* ^2^)] = 0.042
*wR*(*F* ^2^) = 0.116
*S* = 1.05
5101 reflections
349 parameters
H atoms treated by a mixture of independent and constrained refinement
Δρ_max_ = 0.43 e Å^−3^
Δρ_min_ = −0.43 e Å^−3^

### 

Data collection: *APEX2* (Bruker, 2007 ▶); cell refinement: *SAINT* (Bruker, 2007 ▶); data reduction: *SAINT*; program(s) used to solve structure: *SHELXS97* (Sheldrick, 2008 ▶); program(s) used to refine structure: *SHELXL97* (Sheldrick, 2008 ▶); molecular graphics: *ORTEP-3 for Windows* (Farrugia, 2012 ▶); software used to prepare material for publication: *WinGX* (Farrugia, 2012 ▶) and *PLATON* (Spek, 2009 ▶).

## Supplementary Material

Crystal structure: contains datablock(s) I, global. DOI: 10.1107/S1600536813031097/xu5750sup1.cif

Structure factors: contains datablock(s) I. DOI: 10.1107/S1600536813031097/xu5750Isup2.hkl

Additional supplementary materials:  crystallographic information; 3D view; checkCIF report

## Figures and Tables

**Table 1:** Hydrogen-bond geometry (Å, °) *Cg*2 and *Cg*5 are the centroids of the C4*A*/C5*A*/C8*A*/N9/C9*A* and C20–C25 rings, respectively.

*D*—H⋯*A*	*D*—H	H⋯*A*	*D*⋯*A*	*D*—H⋯*A*
N9—H9⋯O5^i^	0.84 (2)	2.18 (2)	2.9561 (19)	154.1 (18)
C21—H21⋯O4^ii^	0.95	2.38	3.230 (2)	148
C8—H8⋯*Cg*5^iii^	0.95	2.81	3.5127 (18)	131
C10—H10*B*⋯*Cg*2^iv^	0.99	2.58	3.5538 (18)	168

Symmetry codes: (i) 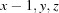; (ii) 
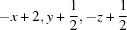
; (iii) 
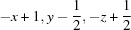
; (iv) 
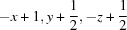
.

**Fig. 1.**
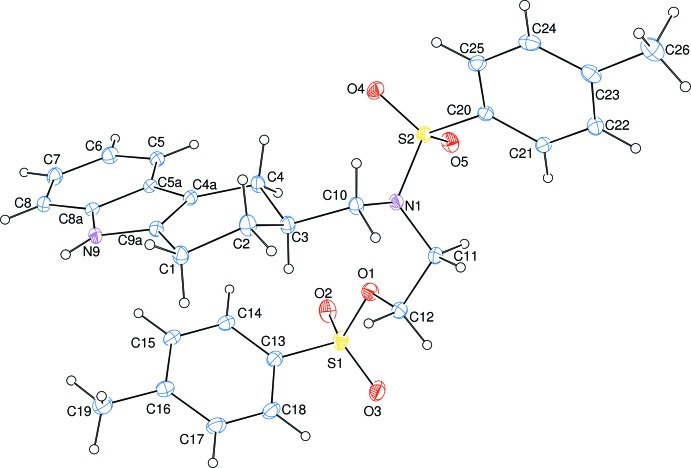
The molecular structure of the title molecule with the atom-numbering scheme. Displacement ellipsoids are drawn at the 50% probability level.

**Fig. 2.**
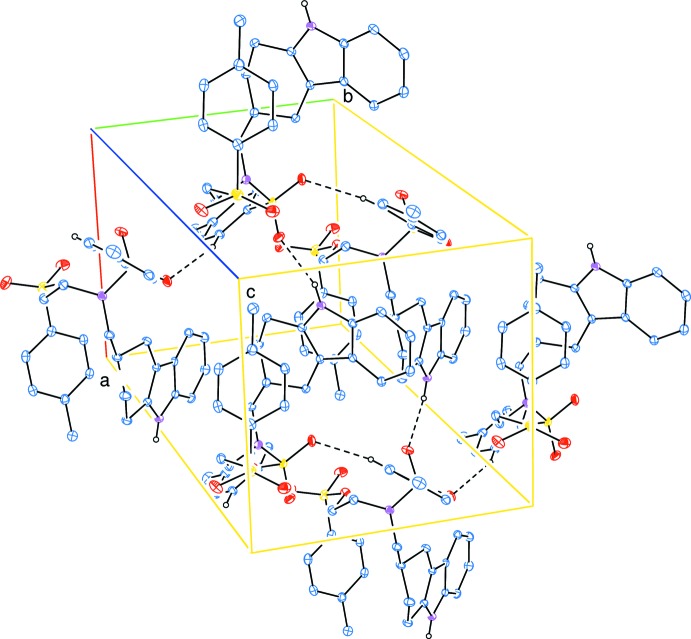
A view of the crystal packing of the title compound. The N—H···O and C—H···O hydrogen bonds are shown as dashed lines [H-atoms not involved in hydrogen bonding have been omitted for clarity].

## supplementary crystallographic information

### 1. Comment

Tetrahydrocarbazole systems are present in the framework of a number of indole-type alkaloids of biological interest (Saxton, 1983). The structures of tricyclic, tetracyclic and pentacyclic ring systems with dithiolane and other substituents of the tetrahydrocarbazole core, have been reported previously. Tetrahydrocarbazoles have been increasingly important intermediates in the syntheses of indole or carbazole alkaloids and various biologically active heterocyclic compounds because of their unique structures. For instance, tetrahydrocarbazole was used in the syntheses of central nervous system active drugs (Romeo *et al.*, 2006). Tetrahydrocarbazolone based antitumor active compounds were synthesized from tetrahydrocarbazoles (Chen *et al.*, 2009). Aminotetrahydrocarbazoles were also synthesized as central nervous system agents (Mooradian *et al.*, 1977). The present study was undertaken to ascertain the crystal structure of the title compound.

The molecule of the title compound contains a carbazole skeleton with methyl phenylsulfonamide and ethyl methyl benzenesulfonate groups, (Fig. 1), where the bond lengths are close to standard values (Allen *et al.*, 1987) and generally agree with those in the previously reported compounds. In all structures atom N9 is substituted.

An examination of the deviations from the least-squares planes through individual rings shows that rings *B* (C4a/C5a/C8a/N9/C9a) and *C* (C5a/C5—C8/C8a) are nearly coplanar [with a maximum deviation of 0.013 (2) Å for atom C7] with dihedral angle of *B*/*C* = 0.85 (6)°. Ring *A* (C1—C4/C4a/C9a) adopts envelope conformation with atom C3 displaced by -0.605 (2) Å from the plane of the other rings atoms, as in 3a,4,10,10b-tetrahydro-2*H*-furo[2,3-a]carbazol-5(3H)-one (Çaylak *et al.*, 2007), 3,3-ethylenedithio-3,3a,4,5,10,10b-hexahydro-2*H*-furo[2,3-a]carbazole (Uludağ *et al.*, 2009), ethyl 1-oxo-1,2,3,4-tetrahydro-9*H*-carbazole-3-carboxylate (Hökelek *et al.*, 2009) and ethyl 4-oxo-2,3,4,9-tetrahydro-1*H*-carbazole-3-carboxylate (Gündoğdu *et al.*, 2011). Ring *A* has a pseudo twofold axis running through the midpoints of C2–C3 and C4a–C9a bonds. Rings *D* (C13—C18) and *E* (C20—C25) are oriented at a dihedral angle of 89.09 (8)°.

In the crystal, intermolecular N—H···O and C—H···O hydrogen bonds link the molecules into infinite chains along the a-axis (Table 1 and Fig. 2). π···π contacts between the benzene rings, Cg4—Cg4^i^ [symmetry code: (i) 1 - *x*, 1 - *y*, 1 - *z*, where Cg4 is the centroid of the ring *D* (C13—C18)] may further stabilize the structure, with centroid-centroid distance of 3.955 (1) Å. There also exist two weak C—H···π interactions (Table 1).

### 2. Experimental

For the preparation of the title compound, (I), a solution of 2-((2,3,4,9 -tetrahydro-1*H*-carbazole-3-yl)methylamino)ethanol (1.0 g, 4.1 mmol) in pyridine (5 ml) was cooled to 273 K. Then, p-toluenesulphonyl chloride (1.7 g, 9.0 mmol) was added. The mixture was stirred for 18 h at room temperature, and then washed with hydrochloric acid (10%). The organic layer was extracted with chloroform and dried over anhydrous magnesium sulfate. The solvent was removed under reduced pressure. The crude product was purified by silica gel column chromatography eluting with ethyl acetate:hexane (1:1). The solvent was evaporated under reduced pressure and the residue was recrystallized from methanol (yield; 1.7 g, 75%, m.p. 425 K).

### 3. Refinement

The H9 atom is located in a difference Fourier synthesis and refined isotropically. The remaining C-bound H-atoms were positioned geometrically with C—H = 0.95, 1.00, 0.99 and 0.98 Å, for aromatic, methine, methylene and methyl H-atoms, respectively, and constrained to ride on their parent atoms, with *U*_iso_(H) = k × *U*_eq_(C), where k = 1.5 for methyl H-atoms and k = 1.2 for all other H-atoms.

### Crystal data

**Table:**

C_29_H_32_N_2_O_5_S_2_	*F*(000) = 1168
*M**_r_* = 552.69	*D*_x_ = 1.339 Mg m^−^^3^
Monoclinic, *P*2_1_/*c*	Cu *K*α radiation, λ = 1.54178 Å
Hall symbol: -P 2ybc	Cell parameters from 9835 reflections
*a* = 10.5719 (5) Å	θ = 3.7–69.1°
*b* = 10.8783 (6) Å	µ = 2.11 mm^−^^1^
*c* = 23.8868 (11) Å	*T* = 150 K
β = 93.507 (2)°	Block, pink
*V* = 2741.9 (2) Å^3^	0.21 × 0.16 × 0.13 mm
*Z* = 4	

### Data collection

**Table:**

Bruker Kappa APEXII CCD area-detector diffractometer	5101 independent reflections
Radiation source: fine-focus sealed tube	4955 reflections with *I* > 2σ(*I*)
Graphite monochromator	*R*_int_ = 0.048
φ and ω scans	θ_max_ = 69.3°, θ_min_ = 3.7°
Absorption correction: multi-scan (*SADABS*; Bruker, 2005)	*h* = −12→12
*T*_min_ = 0.701, *T*_max_ = 0.761	*k* = −12→9
168526 measured reflections	*l* = −28→28

### Refinement

**Table:**

Refinement on *F*^2^	Primary atom site location: structure-invariant direct methods
Least-squares matrix: full	Secondary atom site location: difference Fourier map
*R*[*F*^2^ > 2σ(*F*^2^)] = 0.042	Hydrogen site location: inferred from neighbouring sites
*wR*(*F*^2^) = 0.116	H atoms treated by a mixture of independent and constrained refinement
*S* = 1.05	*w* = 1/[σ^2^(*F*_o_^2^) + (0.0702*P*)^2^ + 1.3803*P*] where *P* = (*F*_o_^2^ + 2*F*_c_^2^)/3
5101 reflections	(Δ/σ)_max_ = 0.001
349 parameters	Δρ_max_ = 0.43 e Å^−^^3^
0 restraints	Δρ_min_ = −0.43 e Å^−^^3^

### Special details

**Table:**

Geometry. All esds (except the esd in the dihedral angle between two l.s. planes) are estimated using the full covariance matrix. The cell esds are taken into account individually in the estimation of esds in distances, angles and torsion angles; correlations between esds in cell parameters are only used when they are defined by crystal symmetry. An approximate (isotropic) treatment of cell esds is used for estimating esds involving l.s. planes.
Refinement. Refinement of F^2^ against ALL reflections. The weighted R-factor wR and goodness of fit S are based on F^2^, conventional R-factors R are based on F, with F set to zero for negative F^2^. The threshold expression of F^2^ > 2sigma(F^2^) is used only for calculating R-factors(gt) etc. and is not relevant to the choice of reflections for refinement. R-factors based on F^2^ are statistically about twice as large as those based on F, and R- factors based on ALL data will be even larger.

### Fractional atomic coordinates and isotropic or equivalent isotropic displacement parameters (Å^2^)

**Table:**

	*x*	*y*	*z*	*U*_iso_*/*U*_eq_	
S1	0.74839 (4)	0.45706 (4)	0.440644 (18)	0.03720 (14)	
S2	0.91673 (4)	0.22863 (4)	0.250089 (17)	0.03013 (13)	
O1	0.79745 (12)	0.41437 (12)	0.38233 (5)	0.0376 (3)	
O2	0.79715 (13)	0.36599 (15)	0.47877 (6)	0.0483 (4)	
O3	0.77959 (14)	0.58320 (14)	0.44931 (6)	0.0477 (3)	
O4	0.85386 (12)	0.11380 (11)	0.23933 (6)	0.0414 (3)	
O5	1.01947 (11)	0.23485 (13)	0.29196 (6)	0.0401 (3)	
N1	0.81003 (12)	0.32611 (12)	0.26881 (6)	0.0279 (3)	
N9	0.26066 (13)	0.12277 (12)	0.33533 (6)	0.0274 (3)	
H9	0.183 (2)	0.1403 (18)	0.3313 (8)	0.029 (5)*	
C1	0.33412 (15)	0.27199 (16)	0.26254 (7)	0.0323 (4)	
H1A	0.2622	0.2488	0.2362	0.039*	
H1B	0.3134	0.3508	0.2806	0.039*	
C2	0.45468 (16)	0.28622 (18)	0.23078 (8)	0.0364 (4)	
H2A	0.4602	0.2173	0.2040	0.044*	
H2B	0.4497	0.3636	0.2090	0.044*	
C3	0.57387 (15)	0.28803 (15)	0.26999 (7)	0.0309 (4)	
H3	0.5629	0.3536	0.2986	0.037*	
C4	0.59305 (14)	0.16623 (15)	0.30117 (7)	0.0289 (3)	
H4A	0.6225	0.1027	0.2753	0.035*	
H4B	0.6585	0.1761	0.3323	0.035*	
C4A	0.47025 (14)	0.12658 (14)	0.32400 (6)	0.0256 (3)	
C5	0.52340 (16)	−0.03680 (15)	0.40220 (7)	0.0297 (3)	
H5	0.6127	−0.0374	0.3996	0.036*	
C5A	0.44685 (15)	0.04014 (13)	0.36752 (6)	0.0252 (3)	
C6	0.46753 (17)	−0.11183 (16)	0.44023 (7)	0.0358 (4)	
H6	0.5192	−0.1639	0.4640	0.043*	
C7	0.33587 (17)	−0.11239 (17)	0.44440 (8)	0.0365 (4)	
H7	0.2996	−0.1660	0.4704	0.044*	
C8	0.25776 (16)	−0.03621 (15)	0.41124 (7)	0.0314 (4)	
H8	0.1686	−0.0359	0.4144	0.038*	
C8A	0.31414 (15)	0.03974 (14)	0.37326 (6)	0.0258 (3)	
C9A	0.35577 (14)	0.17487 (14)	0.30590 (7)	0.0268 (3)	
C10	0.68646 (14)	0.32474 (15)	0.23591 (7)	0.0292 (3)	
H10A	0.6918	0.2666	0.2043	0.035*	
H10B	0.6703	0.4076	0.2199	0.035*	
C11	0.85600 (16)	0.44698 (15)	0.28894 (7)	0.0308 (3)	
H11A	0.8517	0.5055	0.2571	0.037*	
H11B	0.9460	0.4394	0.3026	0.037*	
C12	0.78106 (16)	0.49826 (16)	0.33539 (7)	0.0324 (4)	
H12A	0.8126	0.5811	0.3463	0.039*	
H12B	0.6903	0.5048	0.3229	0.039*	
C13	0.58318 (17)	0.44288 (16)	0.43154 (7)	0.0321 (4)	
C14	0.52784 (17)	0.32846 (16)	0.43769 (7)	0.0328 (4)	
H14	0.5789	0.2582	0.4462	0.039*	
C15	0.39759 (17)	0.31795 (16)	0.43132 (7)	0.0332 (4)	
H15	0.3594	0.2399	0.4361	0.040*	
C16	0.32127 (17)	0.41895 (16)	0.41813 (7)	0.0344 (4)	
C17	0.37962 (19)	0.53315 (17)	0.41211 (8)	0.0411 (4)	
H17	0.3288	0.6033	0.4032	0.049*	
C18	0.50931 (19)	0.54588 (16)	0.41887 (8)	0.0393 (4)	
H18	0.5477	0.6241	0.4149	0.047*	
C19	0.17940 (19)	0.40483 (19)	0.41047 (9)	0.0445 (4)	
H19A	0.1532	0.4090	0.3704	0.067*	
H19B	0.1545	0.3252	0.4256	0.067*	
H19C	0.1383	0.4711	0.4304	0.067*	
C20	0.97518 (15)	0.27802 (14)	0.18609 (7)	0.0283 (3)	
C21	1.05368 (15)	0.38076 (15)	0.18504 (7)	0.0305 (3)	
H21	1.0743	0.4261	0.2183	0.037*	
C22	1.10105 (16)	0.41571 (17)	0.13478 (8)	0.0346 (4)	
H22	1.1539	0.4862	0.1338	0.041*	
C23	1.07314 (16)	0.34982 (18)	0.08539 (7)	0.0363 (4)	
C24	0.99322 (17)	0.24845 (17)	0.08763 (8)	0.0374 (4)	
H24	0.9725	0.2028	0.0544	0.045*	
C25	0.94355 (16)	0.21302 (16)	0.13735 (8)	0.0338 (4)	
H25	0.8880	0.1445	0.1381	0.041*	
C26	1.1295 (2)	0.3854 (2)	0.03154 (9)	0.0509 (5)	
H26A	1.0676	0.3703	0.0000	0.076*	
H26B	1.2058	0.3363	0.0267	0.076*	
H26C	1.1518	0.4728	0.0327	0.076*	

### Atomic displacement parameters (Å^2^)

**Table:**

	*U*^11^	*U*^22^	*U*^33^	*U*^12^	*U*^13^	*U*^23^
S1	0.0384 (2)	0.0419 (3)	0.0316 (2)	−0.00848 (18)	0.00467 (17)	0.00134 (17)
S2	0.0248 (2)	0.0244 (2)	0.0415 (2)	0.00333 (14)	0.00396 (16)	0.00732 (15)
O1	0.0419 (7)	0.0361 (7)	0.0354 (6)	0.0008 (5)	0.0079 (5)	0.0068 (5)
O2	0.0413 (7)	0.0644 (9)	0.0388 (7)	−0.0054 (6)	−0.0007 (6)	0.0135 (6)
O3	0.0521 (8)	0.0474 (8)	0.0444 (7)	−0.0204 (7)	0.0113 (6)	−0.0087 (6)
O4	0.0390 (7)	0.0218 (6)	0.0642 (8)	0.0015 (5)	0.0102 (6)	0.0080 (5)
O5	0.0288 (6)	0.0466 (8)	0.0446 (7)	0.0086 (5)	0.0008 (5)	0.0120 (6)
N1	0.0234 (6)	0.0258 (7)	0.0348 (7)	0.0009 (5)	0.0039 (5)	0.0044 (5)
N9	0.0202 (6)	0.0273 (7)	0.0347 (7)	−0.0013 (5)	0.0028 (5)	0.0003 (5)
C1	0.0244 (8)	0.0316 (9)	0.0406 (9)	0.0017 (6)	0.0001 (7)	0.0076 (7)
C2	0.0299 (9)	0.0392 (10)	0.0399 (9)	0.0004 (7)	0.0012 (7)	0.0130 (7)
C3	0.0254 (8)	0.0277 (8)	0.0397 (9)	0.0013 (6)	0.0035 (6)	0.0087 (7)
C4	0.0231 (7)	0.0272 (8)	0.0367 (8)	0.0014 (6)	0.0032 (6)	0.0078 (6)
C4A	0.0251 (7)	0.0213 (7)	0.0305 (8)	−0.0009 (6)	0.0022 (6)	0.0014 (6)
C5	0.0291 (8)	0.0261 (8)	0.0342 (8)	0.0000 (6)	0.0032 (6)	0.0017 (6)
C5A	0.0263 (8)	0.0203 (7)	0.0292 (8)	−0.0031 (6)	0.0041 (6)	−0.0023 (6)
C6	0.0398 (9)	0.0306 (9)	0.0370 (9)	0.0007 (7)	0.0024 (7)	0.0077 (7)
C7	0.0417 (10)	0.0322 (9)	0.0365 (9)	−0.0075 (7)	0.0092 (7)	0.0056 (7)
C8	0.0294 (8)	0.0302 (8)	0.0351 (9)	−0.0067 (6)	0.0070 (7)	−0.0034 (6)
C8A	0.0266 (8)	0.0217 (7)	0.0294 (8)	−0.0024 (6)	0.0030 (6)	−0.0049 (6)
C9A	0.0237 (7)	0.0252 (8)	0.0317 (8)	−0.0020 (6)	0.0020 (6)	−0.0002 (6)
C10	0.0250 (8)	0.0292 (8)	0.0336 (8)	0.0012 (6)	0.0025 (6)	0.0076 (6)
C11	0.0299 (8)	0.0283 (8)	0.0347 (8)	−0.0031 (6)	0.0063 (6)	0.0035 (6)
C12	0.0338 (8)	0.0286 (8)	0.0350 (8)	−0.0012 (7)	0.0046 (7)	0.0037 (7)
C13	0.0396 (9)	0.0304 (8)	0.0267 (8)	−0.0045 (7)	0.0053 (7)	−0.0013 (6)
C14	0.0405 (9)	0.0257 (8)	0.0323 (8)	0.0002 (7)	0.0024 (7)	0.0004 (6)
C15	0.0422 (9)	0.0247 (8)	0.0325 (8)	−0.0039 (7)	0.0008 (7)	−0.0021 (6)
C16	0.0408 (9)	0.0308 (9)	0.0314 (8)	0.0003 (7)	0.0006 (7)	−0.0035 (7)
C17	0.0469 (11)	0.0262 (9)	0.0500 (11)	0.0044 (7)	0.0014 (8)	0.0023 (7)
C18	0.0503 (11)	0.0253 (9)	0.0428 (10)	−0.0047 (7)	0.0074 (8)	0.0013 (7)
C19	0.0429 (10)	0.0385 (10)	0.0513 (11)	0.0006 (8)	−0.0037 (8)	−0.0032 (8)
C20	0.0227 (7)	0.0241 (8)	0.0382 (9)	0.0043 (6)	0.0032 (6)	0.0000 (6)
C21	0.0270 (8)	0.0272 (8)	0.0375 (9)	−0.0006 (6)	0.0031 (6)	−0.0041 (6)
C22	0.0288 (8)	0.0327 (9)	0.0428 (9)	−0.0009 (7)	0.0070 (7)	0.0011 (7)
C23	0.0312 (8)	0.0413 (10)	0.0367 (9)	0.0114 (7)	0.0052 (7)	0.0007 (7)
C24	0.0337 (9)	0.0375 (9)	0.0404 (9)	0.0098 (7)	−0.0010 (7)	−0.0104 (8)
C25	0.0274 (8)	0.0263 (8)	0.0475 (10)	0.0029 (6)	0.0007 (7)	−0.0063 (7)
C26	0.0508 (12)	0.0624 (14)	0.0406 (10)	0.0102 (10)	0.0119 (9)	0.0045 (9)

### Geometric parameters (Å, º)

**Table:**

S1—O1	1.5854 (13)	C9A—C1	1.487 (2)
S1—O2	1.4209 (15)	C9A—C4A	1.365 (2)
S1—O3	1.4234 (15)	C10—H10A	0.9900
S1—C13	1.7536 (18)	C10—H10B	0.9900
S2—O4	1.4308 (13)	C11—C12	1.509 (2)
S2—O5	1.4325 (13)	C11—H11A	0.9900
S2—N1	1.6305 (14)	C11—H11B	0.9900
S2—C20	1.7672 (17)	C12—H12A	0.9900
O1—C12	1.448 (2)	C12—H12B	0.9900
N1—C10	1.483 (2)	C13—C14	1.387 (2)
N1—C11	1.472 (2)	C13—C18	1.388 (3)
N9—C8A	1.376 (2)	C14—C15	1.381 (3)
N9—C9A	1.383 (2)	C14—H14	0.9500
N9—H9	0.84 (2)	C15—C16	1.388 (3)
C1—C2	1.530 (2)	C15—H15	0.9500
C1—H1A	0.9900	C16—C17	1.398 (3)
C1—H1B	0.9900	C16—C19	1.508 (3)
C2—C3	1.523 (2)	C17—C18	1.378 (3)
C2—H2A	0.9900	C17—H17	0.9500
C2—H2B	0.9900	C18—H18	0.9500
C3—C4	1.527 (2)	C19—H19A	0.9800
C3—C10	1.536 (2)	C19—H19B	0.9800
C3—H3	1.0000	C19—H19C	0.9800
C4—C4A	1.502 (2)	C20—C21	1.393 (2)
C4—H4A	0.9900	C20—C25	1.385 (2)
C4—H4B	0.9900	C21—C22	1.382 (2)
C5—C6	1.381 (2)	C21—H21	0.9500
C5—H5	0.9500	C22—C23	1.396 (3)
C5A—C4A	1.434 (2)	C22—H22	0.9500
C5A—C5	1.400 (2)	C23—C24	1.392 (3)
C6—C7	1.401 (3)	C23—C26	1.501 (3)
C6—H6	0.9500	C24—C25	1.382 (3)
C7—C8	1.384 (3)	C24—H24	0.9500
C7—H7	0.9500	C25—H25	0.9500
C8—H8	0.9500	C26—H26A	0.9800
C8A—C5A	1.418 (2)	C26—H26B	0.9800
C8A—C8	1.388 (2)	C26—H26C	0.9800
			
O1—S1—C13	104.01 (7)	N1—C10—C3	114.07 (13)
O2—S1—O1	103.50 (8)	N1—C10—H10A	108.7
O2—S1—O3	120.48 (9)	N1—C10—H10B	108.7
O2—S1—C13	109.74 (8)	C3—C10—H10A	108.7
O3—S1—O1	108.95 (8)	C3—C10—H10B	108.7
O3—S1—C13	108.84 (9)	H10A—C10—H10B	107.6
O4—S2—O5	119.39 (8)	N1—C11—C12	113.12 (13)
O4—S2—N1	107.19 (7)	N1—C11—H11A	109.0
O4—S2—C20	107.05 (8)	N1—C11—H11B	109.0
O5—S2—N1	106.59 (8)	C12—C11—H11A	109.0
O5—S2—C20	107.42 (8)	C12—C11—H11B	109.0
N1—S2—C20	108.90 (7)	H11A—C11—H11B	107.8
C12—O1—S1	117.76 (11)	O1—C12—C11	107.04 (14)
C10—N1—S2	116.74 (11)	O1—C12—H12A	110.3
C11—N1—S2	116.77 (10)	O1—C12—H12B	110.3
C11—N1—C10	116.39 (13)	C11—C12—H12A	110.3
C8A—N9—C9A	108.75 (13)	C11—C12—H12B	110.3
C8A—N9—H9	125.7 (13)	H12A—C12—H12B	108.6
C9A—N9—H9	125.5 (13)	C14—C13—S1	119.29 (14)
C2—C1—H1A	110.0	C14—C13—C18	120.80 (17)
C2—C1—H1B	110.0	C18—C13—S1	119.91 (14)
C9A—C1—C2	108.65 (13)	C13—C14—H14	120.4
C9A—C1—H1A	110.0	C15—C14—C13	119.13 (16)
C9A—C1—H1B	110.0	C15—C14—H14	120.4
H1B—C1—H1A	108.3	C14—C15—C16	121.43 (16)
C1—C2—H2A	109.1	C14—C15—H15	119.3
C1—C2—H2B	109.1	C16—C15—H15	119.3
C3—C2—C1	112.28 (14)	C15—C16—C17	118.20 (17)
C3—C2—H2A	109.1	C15—C16—C19	120.47 (16)
C3—C2—H2B	109.1	C17—C16—C19	121.33 (17)
H2A—C2—H2B	107.9	C16—C17—H17	119.4
C2—C3—C4	111.63 (14)	C18—C17—C16	121.29 (17)
C2—C3—C10	108.43 (14)	C18—C17—H17	119.4
C2—C3—H3	107.7	C13—C18—H18	120.4
C4—C3—C10	113.59 (13)	C17—C18—C13	119.14 (17)
C4—C3—H3	107.7	C17—C18—H18	120.4
C10—C3—H3	107.7	C16—C19—H19C	109.5
C3—C4—H4A	109.8	C16—C19—H19B	109.5
C3—C4—H4B	109.8	C16—C19—H19A	109.5
C4A—C4—C3	109.53 (13)	H19C—C19—H19B	109.5
C4A—C4—H4A	109.8	H19C—C19—H19A	109.5
C4A—C4—H4B	109.8	H19B—C19—H19A	109.5
H4A—C4—H4B	108.2	C21—C20—S2	119.98 (13)
C5A—C4A—C4	129.99 (14)	C25—C20—S2	119.51 (13)
C9A—C4A—C4	122.95 (14)	C25—C20—C21	120.51 (16)
C9A—C4A—C5A	107.03 (13)	C20—C21—H21	120.5
C5A—C5—H5	120.4	C22—C21—C20	118.96 (16)
C6—C5—C5A	119.20 (15)	C22—C21—H21	120.5
C6—C5—H5	120.4	C21—C22—C23	121.57 (17)
C5—C5A—C4A	134.57 (14)	C21—C22—H22	119.2
C5—C5A—C8A	118.71 (14)	C23—C22—H22	119.2
C8A—C5A—C4A	106.72 (13)	C22—C23—C26	121.15 (18)
C5—C6—C7	121.07 (16)	C24—C23—C22	118.13 (16)
C5—C6—H6	119.5	C24—C23—C26	120.71 (18)
C7—C6—H6	119.5	C23—C24—H24	119.4
C6—C7—H7	119.4	C25—C24—C23	121.14 (16)
C8—C7—C6	121.13 (15)	C25—C24—H24	119.4
C8—C7—H7	119.4	C20—C25—H25	120.2
C7—C8—C8A	117.73 (15)	C24—C25—C20	119.66 (16)
C7—C8—H8	121.1	C24—C25—H25	120.2
C8A—C8—H8	121.1	C23—C26—H26B	109.5
N9—C8A—C5A	107.66 (13)	C23—C26—H26C	109.5
N9—C8A—C8	130.19 (15)	C23—C26—H26A	109.5
C8—C8A—C5A	122.14 (15)	H26B—C26—H26A	109.5
N9—C9A—C1	124.01 (14)	H26B—C26—H26C	109.5
C4A—C9A—N9	109.84 (14)	H26C—C26—H26A	109.5
C4A—C9A—C1	126.12 (14)		
			
O2—S1—O1—C12	−170.46 (12)	C5—C5A—C4A—C9A	−179.36 (17)
O3—S1—O1—C12	−41.11 (15)	C8A—C5A—C4A—C4	178.54 (16)
C13—S1—O1—C12	74.85 (13)	C8A—C5A—C4A—C9A	0.63 (17)
O1—S1—C13—C14	81.47 (14)	C4A—C5A—C5—C6	−179.06 (17)
O1—S1—C13—C18	−99.17 (15)	C8A—C5A—C5—C6	1.0 (2)
O2—S1—C13—C14	−28.72 (16)	C5—C6—C7—C8	−1.2 (3)
O2—S1—C13—C18	150.64 (15)	C6—C7—C8—C8A	0.7 (3)
O3—S1—C13—C14	−162.50 (13)	N9—C8A—C5A—C4A	−0.49 (16)
O3—S1—C13—C18	16.86 (17)	N9—C8A—C5A—C5	179.50 (14)
O4—S2—N1—C10	−43.26 (13)	C8—C8A—C5A—C4A	178.62 (14)
O4—S2—N1—C11	172.49 (12)	C8—C8A—C5A—C5	−1.4 (2)
O5—S2—N1—C10	−172.17 (11)	N9—C8A—C8—C7	179.43 (16)
O5—S2—N1—C11	43.58 (13)	C5A—C8A—C8—C7	0.5 (2)
C20—S2—N1—C10	72.23 (12)	N9—C9A—C1—C2	−167.88 (15)
C20—S2—N1—C11	−72.03 (13)	C4A—C9A—C1—C2	14.3 (2)
O4—S2—C20—C21	−172.61 (13)	N9—C9A—C4A—C4	−178.63 (14)
O4—S2—C20—C25	6.62 (15)	N9—C9A—C4A—C5A	−0.54 (18)
O5—S2—C20—C21	−43.26 (15)	C1—C9A—C4A—C4	−0.6 (3)
O5—S2—C20—C25	135.98 (13)	C1—C9A—C4A—C5A	177.54 (15)
N1—S2—C20—C21	71.81 (14)	N1—C11—C12—O1	62.68 (17)
N1—S2—C20—C25	−108.95 (13)	S1—C13—C14—C15	179.03 (13)
S1—O1—C12—C11	169.71 (11)	C18—C13—C14—C15	−0.3 (3)
S2—N1—C10—C3	116.55 (14)	S1—C13—C18—C17	−179.70 (14)
C11—N1—C10—C3	−99.06 (16)	C14—C13—C18—C17	−0.4 (3)
S2—N1—C11—C12	−144.48 (12)	C13—C14—C15—C16	1.0 (3)
C10—N1—C11—C12	71.14 (18)	C14—C15—C16—C17	−0.9 (3)
C9A—N9—C8A—C5A	0.17 (17)	C14—C15—C16—C19	179.01 (16)
C9A—N9—C8A—C8	−178.84 (16)	C15—C16—C17—C18	0.2 (3)
C8A—N9—C9A—C1	−177.89 (15)	C19—C16—C17—C18	−179.71 (18)
C8A—N9—C9A—C4A	0.24 (18)	C16—C17—C18—C13	0.4 (3)
C9A—C1—C2—C3	−44.4 (2)	S2—C20—C21—C22	178.25 (13)
C1—C2—C3—C4	63.82 (19)	C25—C20—C21—C22	−1.0 (2)
C1—C2—C3—C10	−170.31 (14)	S2—C20—C25—C24	−177.40 (13)
C2—C3—C4—C4A	−46.36 (19)	C21—C20—C25—C24	1.8 (2)
C10—C3—C4—C4A	−169.34 (14)	C20—C21—C22—C23	−0.7 (3)
C2—C3—C10—N1	−178.08 (14)	C21—C22—C23—C24	1.4 (3)
C4—C3—C10—N1	−53.4 (2)	C21—C22—C23—C26	−177.40 (17)
C3—C4—C4A—C5A	−161.21 (16)	C22—C23—C24—C25	−0.6 (3)
C3—C4—C4A—C9A	16.4 (2)	C26—C23—C24—C25	178.28 (17)
C5A—C5—C6—C7	0.3 (3)	C23—C24—C25—C20	−1.1 (3)
C5—C5A—C4A—C4	−1.4 (3)		

### Hydrogen-bond geometry (Å, º)

Cg2 and Cg5 are the centroids of the C4A/C5A/C8A/N9/C9A and C20–C25 rings, respectively.

**Table:**

*D*—H···*A*	*D*—H	H···*A*	*D*···*A*	*D*—H···*A*
N9—H9···O5^i^	0.84 (2)	2.18 (2)	2.9561 (19)	154.1 (18)
C21—H21···O4^ii^	0.95	2.38	3.230 (2)	148
C8—H8···*Cg*5^iii^	0.95	2.81	3.5127 (18)	131
C10—H10*B*···*Cg*2^iv^	0.99	2.58	3.5538 (18)	168

Symmetry codes: (i) *x*−1, *y*, *z*; (ii) −*x*+2, *y*+1/2, −*z*+1/2; (iii) −*x*+1, *y*−1/2, −*z*+1/2; (iv) −*x*+1, *y*+1/2, −*z*+1/2.
